# Foreign Body in the Bronchi of an Infant

**Published:** 1844-11-16

**Authors:** 


FOREIGN BODY IN THE BRONCHI OF AN INFANT.

An infant two months old, was on the knee of its mother, who was engaged in cleaning French beans. Suddenly it was seized with a convulsive cough, coming on in fits, with intervals of perfect calm. On examination, the face was found pale, with an expression of suffering; respiration difficult, and attended with lifting of the alæ of the nose. No fever, nor any abnormal sounds on auscultation. At the slightest motion a fit of suffocating cough came on, and the child leaned forward and opened its mouth as if to vomit. Various conjectures were advanced as to the nature of the case. Leeches, blisters, and emetics were had recourse to. It was thought a foreign body might be present, and the trachea was opened, but none was found. On examination of the body after death, there was found at the bifurcation of the bronchi, a bean, surrounded by pus and false membrane. The lungs were hepatized."—*Edin. Monthly Journal.*

